# The altered immunological status of children conceived by assisted reproductive technology

**DOI:** 10.1186/s12958-021-00858-2

**Published:** 2021-11-26

**Authors:** Xin Xu, Han Wu, Yuehong Bian, Linlin Cui, Yuanyuan Man, Zhao Wang, Xin Zhang, Changming Zhang, Ling Geng

**Affiliations:** 1 Center for Reproductive Medicine, Cheeloo College of Medicine, Shandong University, Jinan, 250012 Shandong China; 2 Key laboratory of Reproductive Endocrinology of Ministry of Education, Shandong University, Jinan, 250012 Shandong China; 3 Shandong Key Laboratory of Reproductive Medicine, Jinan, 250012 Shandong China; 4 Shandong Provincial Clinical Research Center for Reproductive Health, Jinan, 250012 Shandong China; 5 Department of Obstetrics and Gynecology, Shandong Provincial Hospital Affiliated to Shandong First Medical University, No.324, Jingwu Road, Jinan, 250021 Shandong China

**Keywords:** Assisted reproductive technology, Children, Immune system, Interleukin-4, Interferon-gamma

## Abstract

**Background:**

With the increased use of assisted reproductive technology (ART), assessing the potential health risks of children conceived on ART important to public health. Most research in this area has focused on the effects of ART on perinatal, metabolic, and oncological risks in children. Although an increased risk of immune-related diseases has been reported in children born after ART, there are no studies on the immunological status of these children. This study aimed to evaluate the impact of different embryo transfer methods and fertilization strategies on the immune status of the offspring.

**Methods:**

A total of 69 children born to women treated with ART and a matched control group of 17 naturally conceived (NC) children, all aged from 3 to 6 years, were recruited in the reproductive hospital affiliated to Shandong University. The frequency of immune cells in the peripheral blood was assayed using flow cytometry; plasma cytokine levels were determined by multiplex cytokine immunoassay with human cytokine magnetic beads.

**Results:**

Compared to children born after natural conception, children born after ART had elevated interferon-γ (IFN-γ) levels, regardless of embryo transfer and fertilization strategies. Children in the fresh-embryo transfer group had significantly higher IL-4 levels and a lower ratio of IFN-γ to IL-4 than those in the NC group ((*P* = 0.004, 10.41 ± 5.76 pg/mL vs 18.40 ± 7.01 pg/mL, *P* = 0.023, 1.00 ± 0.48 vs 0.67 ± 0.32, respectively). Similar results were shown in either the in vitro fertilization (IVF) group or the intra-cytoplasmic sperm injection (ICSI) group (*P* < 0.05 and *P* = 0.08 for IVF; *P* < 0.05 and *P* < 0.05 for ICSI, respectively). These alterations in IL-4 concentrations and the ratio of IFN-γ to IL-4 were statistically significantly correlated with supra-physical E_2_ (estradiol) levels on the day of hCG administration (*R* = 0.502, *P* = 0.017; *R* = − 0.537, *P* = 0.010, respectively). Consistently, the frozen embryo transfer did not result in alterations of these immune indicators in the offspring. Overall, there were no significant differences between the ART group and NC group in the frequencies of T cells, B cells, natural killer (NK) cells, CD4^+^T cells, CD8^+^T cells, T helper (T_H_)1 cells, T_H_17 cells, and regulatory T (T_reg_) cells and cytokine levels of IL-10 and IL-17a (all *P* > 0.05).

**Conclusions:**

Immunological alterations existed in children born after the use of ART. The elevated E_2_ levels before embryo implantation contributed to the increased IL-4 levels in children conceived by fresh embryo transfer. The assessment of immunological alteration is of importance to children conceived by ART for early monitoring and intervention.

**Supplementary Information:**

The online version contains supplementary material available at 10.1186/s12958-021-00858-2.

## Introduction

Since 1978, more than 8 million children have been born globally after assisted reproductive technology (ART) treatment [1]. When compared with natural conception (NC), in vitro fertilization (IVF) and intra-cytoplasmic sperm injection (ICSI) involves several artificial procedures, such as ovary stimulation and the in vitro manipulation of gametes and embryos. Based on the theory of the developmental origins of health and disease, children conceived using these interventions may face an increased risk for adverse perinatal outcomes [2, 3] and chronic immunological disorders in later life [4].

Alterations to children’s immune systems after ART treatment have gained increasing traction in animal and human studies. ART conceived mice exhibited less efficient immune responses to vaccines and promoted T helper (T_H_) 2 immune responses [5, 6]. ART conceived children were associated with a high prevalence of immune-related diseases, including metabolic disorders and allergic diseases [7–11]. Also, immune response gene expression was altered in the placenta from patients undergoing IVF treatment [12], potentially affecting offspring immune responses [13]. In addition, ART pregnancies were linked to increased tumor risk, which potentially suggested increased immune tolerance to tumor antigens [10]. These animal studies and clinical observations imply that ART treatment may affect the immune profile of offspring.

CD4 + T helper (T_H_) cells modulate and orchestrate the function of other immune cells and play central roles in the adaptive immune system [14]. Effector CD4 + T cells, differentiated from naïve CD4+ T cells, are functionally classified into four principal lineages, T_H_1, T_H_2, T_H_17, and T regulatory (Treg), based on the expression of characteristically expressed cytokines and transcription factors [15]. Any imbalance of these indicators may predispose the individuals to various pathological conditions and immune dysfunctions [16]. Therefore, the assessment of immunological alteration is vital to children conceived by ART for early monitoring and intervention.

Therefore, in this retrospective study, we examined TH cells frequencies and associated cytokine levels in the peripheral blood of children born after ART and natural conception. We intend to determine whether offspring conceived by ART have immunological alterations in childhood.

## Materials and methods

### Study population

All children were recruited from a retrospective and prospective birth cohort at the Center for Reproductive Medicine, Shandong University, China. The children aged 3-6 years were invited for medical examination. Seventeen children in the control group were born by natural conception, whose mothers had not received any ART treatment before these offspring’s birth. This was further confirmed through face-to-face interviews. The ART group comprised 69 children born to mothers received ART, who were randomly selected, except for exclusion criteria. One of the twins was randomized to our study. Mothers with a history of recurrent spontaneous abortion, premature ovarian insufficiency, autoimmune diseases, infectious diseases were excluded.

### Collection of clinical information

Pre-pregnancy information on parents, e.g. smoking history, education, and parity, were collected at study commencement. Data on infant plurality, gestational weeks, and birth weight was collected via telephone follow-up or face-to-face interview. Children’s height and body weight were measured using a stadiometer and a calibrated electronic scale, and body mass index (BMI) (kg·m^− 2^) was calculated.

### Lymphocyte isolation

Blood samples from children were collected in the morning (8:00–10:00) after overnight fasting. Peripheral blood mononuclear cells (PBMCs) were isolated from blood using Ficoll-Hypaque density gradient centrifugation. Briefly, whole blood was diluted 1:1 in phosphate-buffered saline (PBS, PH 7.2-7.6). This volume was then added to the same volume of lymphocyte separation medium (MP Biomedicals, USA) and centrifuged for 20 min at 500 g at room temperature. PBMC interface cells were carefully collected and washed twice in PBS. After separation, PBMCs were used for subsequent flow cytometry analysis.

### Flow cytometry

To analyze cell surface markers, PBMCs were stained with monoclonal antibodies directly conjugated to different fluorochromes in staining buffer (PBS plus 1% fetal bovine serum (FBS)) for 20 min at 4 °C in the dark. PBMCs were stimulated using 12-phorbol myristate 13-acetate, ionomycin, brefeldin A and monensin (eBioscience, USA) at 37 °C in 5% CO_2_ for 4 h. Then cells were fixed and permeabilized using a Cytofix/ Cytoperm Plus kit (BD Biosciences, USA) and incubated with antibodies for 30 min at room temperature in the dark. For transcription factor analysis, a Foxp3 staining buffer set (eBioscience, USA) was used according to manufacturers’ instructions and incubated with antibodies (Foxp3-PE (eBioscience, USA)). All flow cytometry data were acquired on an LSR Fortessa cell analyzer (BD Biosciences) and analyzed using FlowJo software (BD Biosciences, the USA). (Fig. S1).

### Multiplex immunoassay

Before the assay, blood samples were centrifuged at 500 g for 10 min at 4 °C and stored at − 80 °C. According to manufacturers’ instructions, the plasma cytokine (IFN-γ, IL-4, IL-17a, and IL-10) levels were assayed using the human cytokine magnetic beads multiplex immunoassay (Merck, Germany). Briefly, samples, standards, and quality control reagents were added to a 96-well plate pre-wetted with wash buffer. Next, premixed beads were added to each well and incubated overnight at 4 °C. After washing, a detection antibody solution was added and incubated for 1 h. Then, a streptavidin-phycoerythrin solution was added and incubated for 30 min. After washing and resuspending beads, the plate was processed and analyzed the Median Fluorescent Intensity (MFI) data using Luminex 2000 software. Cytokine concentrations were calculated from a calibration curve by 5-parametric curve fitting using MILLIPLEX analyst 5.1 software (Merck, Germany).

### Statistical analysis

Normal distributions were checked using the Kolmogorov-Smirnov test. Normalized continuous data were expressed as the mean ± standard deviation and compared using the student’s t-test. Non-normal distribution parameters were presented as the median (interquartile ranges) and compared using the Mann-Whitney U-test. Categorical variables were compared using the χ^2^ test. Pearson’s correlation and Spearman’s correlation were used to estimate the association between the immune indicators and clinical characteristics during ART. Statistical analyses were performed using SPSS software version 26 (IBM Corporation, Armonk, NY, USA) or GraphPad Prism version 8.4.0 (GraphPad Software, La Jolla, CA, USA). *P* < 0.05 value was considered statistically significant.

## Results

### Parental and offspring characteristics

In total, 86 children were recruited, including 17 NC children and 69 ART-conceived children. The average age was 4.3 ± 1.2 years (range 3–6 years old). No significant differences were observed for age and BMI between groups. Parental characteristics and perinatal outcomes of all participants are summarized in Table 1. We observed no significant differences in parental age, BMI, education level, or paternal smoking status between groups (all *P* > 0.05). In addition, the perinatal outcomes, including sex, plurality, gestational week, and birth weight, were comparable between groups (all *P* > 0.05).

**Table 1:** Characteristics of the study population

	NC	ART	*P* value
Maternal age, years	28.06 ± 4.87	28.54 ± 3.68	0.66
Maternal BMI, kg/m^2^	23.08 ± 3.73	23.61 ± 3.70	0.61
Maternal educational level, No(%)			0.38
High school or lower	13(76.5%)	45(65.2%)	
College or higher	4(23.5%)	24(34.8%)	
Maternal parity			0.39
Primiparous	15(88.2%)	65(94.2%)	
≥ 1 child	2(11.8%)	4(5.8%)	
Paternal age, years	29.06 ± 4.29	29.37 ± 4.07	0.78
Paternal BMI, kg/m^2^	24.91 ± 3.39	25.34 ± 4.19	0.70
Paternal educational level, No(%)			0.44
High school or lower	13(76.5)%)	46(66.7%)	
College or higher	4(23.5%)	23(33.3%)	
Smoking, No (%)			0.79
NO	11(64.7%)	47(68.1%)	
Yes	6(35.3%)	22(31.9%)	
Child age, years	4.59 ± 1.12	4.19 ± 1.18	0.21
Child BMI, kg/m^2^	16.70 ± 3.22	16.59 ± 1.81	0.85
Child female sex, n(%)	14(82.4%)	49(71.0%)	0.34
Child twin or not, n(%)			0.17
Singleton	16(94.1%)	52(75.4%)	
Twin	1(5.9%)	17(24.6%)	
Child gestational age, n(%)			0.76
< 37 weeks (preterm)	2(11.8%)	6(8.7%)	
37–42 weeks (full-term)	14(82.4%)	61(88.4%)	
> 42 weeks (post-term)	1(5.9%)	2(2.9%)	
Child birthweight, kg	3.51 ± 0.68	3.28 ± 0.68	0.20

Data are expressed as average ± SD or the median (interquartile range)

*BMI* body mass index, *NC* natrural conception, *IVF* in vitro fertilization, *ICSI* intra-cytoplasmic sperm injection

### Comparison of immune indicators in NC and ART conceived children

We investigated multiple lymphocyte subsets in the peripheral blood of NC and ART-conceived children (Table 2). Overall, we observed no differences in total T, B, and NK cell percentages between groups (all *P* > 0.05). For T cell subsets, we found similar frequencies of CD4^+^T cells and CD8^+^T cells as well as comparable proportions of T_H_1 (CD3^+^ CD8^−^ IFN-γ^+^ T) cells, T_H_17 (CD3^+^ CD8^−^ IL-17A^+^ T) cells and T_reg_ (CD3 + CD4^+^ CD25^hi^ Foxp3^+^) cells (all *P* > 0.05). Next, we assessed associated cytokines levels of IFN-γ, IL-4, IL-17a, IL-10 in children born from NC and ART. Intriguingly, children conceived by ART had higher IFN-γand IL-4 levels than those by NC, but this difference did not reach statistical significance (*P* = 0.063 and *P* = 0.062, respectively). There were no differences in IL-17a and IL-10 levels between groups (all *P* > 0.05).

**Table 2:** Frequency of lymphocytes subsets among children conceived natural conception, IVF and ICSI

	NC	ART	*P* value
% T cells (CD3+)	66.31 ± 12.80	68.79 ± 10.15	0.46
% B cells (CD19+)	12.21 ± 6.12	13.26 ± 5.85	0.57
% NK cells (CD16 + CD56+)	15.29 ± 9.39	13.25 ± 6.90	0.38
% CD8 + T cells	22.93 ± 5.80	24.33 ± 5.39	0.40
% CD4+ T cells	33.83 ± 9.03	35.95 ± 7.87	0.41
% TH1 (CD3 + CD8-IFN-γ+)	4.62(3.01, 8.44)	4.65(2.72, 6.86)	0.72
% TH17 (CD3 + CD8-IL-17+)	0.09(0.06, 0.19)	0.15(0.10, 0.25)	0.17
% Treg (CD4 + CD25hi + Foxp3+)	2.47 ± 0.74	2.32 ± 0.84	0.60
IFH-γ (pg/mL)	8.21 ± 1.75	10.85 ± 4.24	0.06
IL-4 (pg/mL)	10.41 ± 5.76	15.32 ± 7.48	0.06
IL-17A (pg/mL)	6.01 ± 2.35	6.77 ± 2.61	0.43
IL-10 (pg/mL)	4.86 ± 0.86	6.32 ± 3.14	0.20

Data are expressed as average ± SD or the median (interquartile range)

*TH1* T helper 1, *TH17* T helper 17, *Treg* regulatory T cells, *INF-γ* interferon gamma, *IL-4* interleukin-4, *IL-17A* interleukin-17A, *IL-10* interleukin-10

### Comparison of immune indicators in children conceived by different embryo transfer timing

**Fig. 1:**
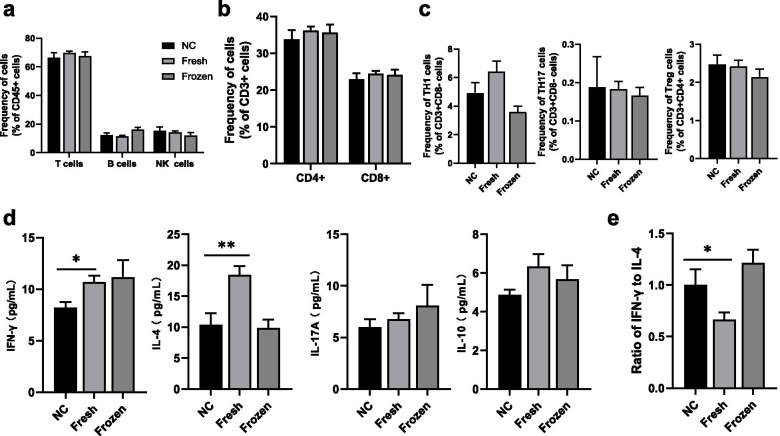
The levels of immune indicators in peripheral blood among children conceived naturally and children conceived by fresh and frozen embryo transfer. **a** the proportion of lymphocyte subsets in flow cytometry. **b** the proportion of CD4+ or CD8+ T cells in flow cytometry. **c** the proportion of TH cell lineage gated from CD3 + CD8- cells (TH1 and TH17) or CD3 + CD4+ (Treg) in flow cytometry. **d** the plasma used for cytokines analysis in multiplex immunoassay. **e** ratio of IFN-γ to IL-4. *P* values were determined by using Student t test or Mann-Whitney U test. Data are presented as means ± SEMs. *P < 0.5, **P < 0.01

The ART group included children born after the use of fresh embryo transfer and frozen embryo transfer. Next, we compared the immunological profile among the fresh embryo transfer group, frozen embryo transfer group, and NC group. We observed no differences in frequencies of total T cells, B cells, and NK cells among the groups (Fig. 1a), similar CD4^+^T cell and CD8^+^T cell proportions (Fig. 1b), and also comparable T_H_1, T_H_17and T_reg_ cell frequencies (Fig. 1c) (all *P* > 0.05). For cytokines, compared with the naturally conceived children, the children born following fresh embryo transfer showed significantly higher IFN-γ levels (*P* = 0.020, 8.21 ± 1.75 pg/mL vs 10.68 ± 2.94 pg/mL) and IL-4 levels(*P* = 0.004, 10.41 ± 5.76 pg/mL vs 18.40 ± 7.01 pg/mL), and lower ratio of IFN-γ to IL-4 (*P* = 0.023, 1.00 ± 0.48 vs 0.67 ± 0.32) (Fig. 1d and e). Likewise, children born after frozen embryo transfer also tended to have elevated IFN-γ levels (*P* = 0.153, 8.21 ± 1.75 pg/mL vs 11.14 ± 6.04 pg/mL) (Fig. 1d). Unexpectedly, we found children conceived frozen embryo transfer had comparable levels of IL-4 and ratio of IFN-γ to IL-4 compared with counterparts of the NC group (all *P* > 0.05) (Fig. 1d and e). There was no significant difference in the levels of IL-10 and IL-17a among groups, respectively (Fig. 1d).

### Comparison of immune indicators in children conceived by IVF and ICSI

**Fig. 2:**
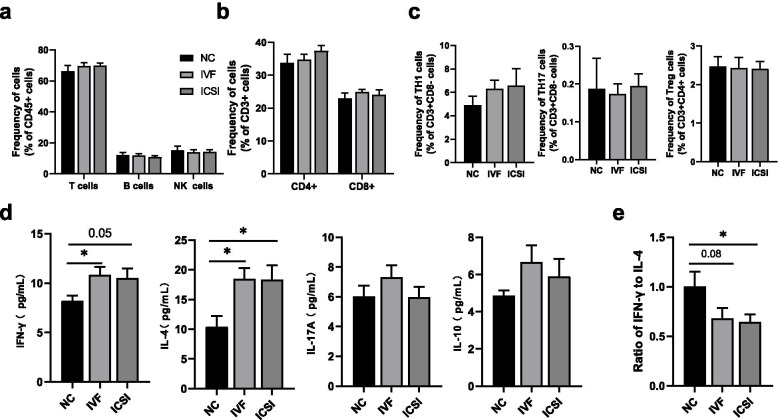
The levels of immune indicators in peripheral blood among naturally conceived children and IVF/ICSI-conceived children. **a** the proportion of lymphocyte subsets in flow cytometry. **b** the proportion of CD4+ or CD8+ T cells in flow cytometry. **c** the proportion of TH cell lineage gated from CD3 + CD8- cells (TH1 and TH17) or CD3 + CD4+ (Treg) in flow cytometry. **d** the plasma used for cytokines analysis in multiplex immunoassay. **e** ratio of IFN-γ to IL-4. *P* values were determined by using Student t test or Mann-Whitney U test. Data are presented as means ± SEMs. *P < 0.5, **P < 0.01

The fresh embryo transfer group included children born after IVF and ICSI. Next, we compared these immune cell frequencies and cytokine levels in children born after IVF or ICSI. We found similar frequencies of T cells, B cells, NK cells (Fig. 2a), CD4 + T cells, CD8 + T cells (Fig. 2b), T_H_1 cells, T_H_17 cells, and T_reg_ cells (Fig. 2c) among three groups. Compared with children in the NC group, those born after IVF showed elevated circulating IFN-γ levels (*P* < 0.05); children conceived by ICSI also had an elevated trend, but this difference did not reach statistical significance(*P* = 0.05). Additionally, we identified higher levels of IL-4 and a lower ratio of IFN-γ to IL-4 in children of the IVF group and the ICSI group (both *P* < 0.05) (Fig. 2d, e). In addition, we observed no significantly different IL-17a and IL-10 levels among groups (Fig. 2d).

### The correlation between immune indicators and clinical characteristics

Next, we conducted a correlation analysis. Interestingly, the amounts of estradiol (E_2_) were positively correlated with the circulating level of IL-4 (*R* = 0.486, *P* = 0.022) but negatively associated with the ratio of IFN-γ to IL-4 (*R* = -0.461, *P* = 0.031). There was no strong correlation between the dose of Gn use, the days of Gn use, and progesterone (P) concentrations and IL-4 or ratio of IFN-γ to IL-4 (all *P* > 0.05) (Table 3).

**Table 3:** Correlation between immune indicators in peripheral blood and ART clinical characteristics

	Gn dose		Gn days		Estradiol		Progesterone	
Viaribles	R	P	R	P	R	P	R	P
% T cells (CD3+)^a^	−0.12	0.53	−0.02	0.94	− 0.17	0.41	− 0.28	0.14
% B cells (CD19+)^a^	0.11	0.56	−0.23	0.25	−0.06	0.76	0.03	0.89
% NK cells (CD16 + CD56+)^a^	0.11	0.57	0.23	0.25	0.26	0.20	0.17	0.37
% CD8 + T cells ^a^	0.27	0.15	−0.09	0.67	−0.03	0.87	−0.07	0.71
% CD4+ T cells ^a^	−0.27	0.15	0.17	0.38	0.10	0.62	−0.32	0.09
% TH1 (CD3 + CD8-IFN-γ+) ^b^	−0.01	0.95	−0.06	0.79	−0.02	0.92	−0.12	0.55
% TH17 (CD3 + CD8-IL-17+) ^b^	−0.15	0.45	0.05	0.81	0.10	0.63	−0.30	0.13
% Treg (CD4 + CD25hi + Foxp3+)^a^	−0.06	0.75	0.04	0.83	0.01	0.97	0.10	0.63
IFH-γ (pg/mL) ^a^	0.32	0.14	−0.29	0.19	− 0.33	0.13	− 0.01	0.98
IL-4 (pg/mL) ^a^	−0.06	0.80	0.49	0.02	0.50	0.02	−0.13	0.56
IFN-γ / IL-4 ^a^	0.16	0.49	−0.46	0.03	−0.54	0.01	0.04	0.86
IL-17A (pg/mL) ^a^	0.34	0.13	0.14	0.56	0.09	0.69	−0.20	0.38
IL-10 (pg/mL) ^a^	0.07	0.75	0.02	0.94	0.01	0.96	−0.01	0.99

Data analyzed by Pearson’s correlations were noted by a and data analyzed by Spearman’s correlations were noted by b

*Abbreviation*: *Gn* Gonadotropin

## Discussion

ART-conceived children face an increased prevalence of metabolic disorders, allergic diseases, and even tumors [8, 10]. However, possible alterations in the immune responses of these children remain unclear. Here, for the first time, we present data on the immune status of children conceived by fresh embryo transfer, frozen embryo transfer, IVF, and ICSI. We found a trend toward elevated pro-inflammatory cytokine IFN-γ in children born to women treated with ART. Compared with children of the NC group, plasma IL-4 levels and the ratio of IFN-γ to IL-4 were significantly different in those conceived by fresh embryo transfer, regardless of fertilization strategies. Intriguingly, the levels of IL-4 and the ratio of IFN-γ to IL-4 significantly correlated with E_2_ levels on the day of hCG administration. Therefore, our data suggested that immunological alterations existed in children conceived by ART and were associated with the E_2_ levels before embryo implantation.

Normal immune homeostasis is critical for supporting and stabilizing an individual’s immune response [14]. Similarly, disturbances in immunocyte and cytokine homeostasis are believed to trigger or mediate immune disorders, including allergies and autoimmune diseases [17–19]. Notably, elevated IL-4 levels exert critical roles in asthma pathogenesis [20]. Allergen challenges induce IL-4 release into the peripheral blood, with IL-4 levels exacerbating peribronchial inflammation causing asthma [18]. Previous two cohort studies have shown that IVF-conceived children have an increased prevalence of asthma and the administration of anti-asthmatic drugs [7–9]. However, another study showed no significant differences in asthma, allergic rhinitis, and atopic dermatitis between the ART group and the control group [21]. One reason may be that children conceived by ART in these studies, including fresh embryo transfer and frozen embryo transfer, but circulating IL-4 levels were similar in children created by frozen embryo transfers and natural conception. Another reason may be the variety of genetic backgrounds. In addition to clinical studies, animal studies identified similar alterations in T_H_ cell-mediated immune responses in ART-conceived mice [5, 6]. Compared with NC mice, the ART group exhibited higher IL-4 serum levels and promoted T_H_2 immune responses in fresh embryo transfer, consistent with our findings. According to these data, we can infer that children conceived by fresh embryo transfer via ART tended to have elevated plasma IL-4 levels and might face a higher risk of allergic diseases.

IFN-γ is a kind of proinflammation cytokine, which plays a critical role in regulating systemic inflammation, insulin resistance, and cardiovascular diseases [22]. Our study observed that children conceived by fresh embryo transfer expressed significantly higher IFN-γ levels than the NC group. Similarly, we found a higher tendency of IFN-γ in children born after ART, IVF, and ICSI, but without significant difference. Mounting evidence showed that IVF treatment might predispose offspring to an adverse metabolic profile and an increased blood pressure in childhood and adolescence [11, 23]. These results implied that children conceived by ART had elevated IFN-γ levels, which might exacerbate metabolic syndrome and cardiovascular disorders.

The underlying mechanisms of immune response alterations remain unclear. Parent-related factors, including infertility, advanced maternal age, and increased risk of pregnancy complications, may also affect the immune systems of offspring [24]. Epigenetic patterns controlling imprinted gene expression are typically established at early gamete and embryo development stages [4]. Altered environmental conditions in the preimplantation period, such as culture media, hormone stimulation drugs, manipulation of gametes and embryos, may lead to epigenetic alterations [23, 25]. Subsequent to these ART treatment, these epigenetic aberrations may alter offspring immune responses and increase susceptibility to immunological problems in later life [12]. Interestingly, we speculated higher IL-4 levels in children conceived by fresh embryo transfer than frozen embryo transfer. This discrepancy implied the role of an unfavorable uterine environment after the fresh-embryo transfer, especially supra-physiological hormonal levels. The strong positive correlations between amounts of inflammatory cytokines IL-4 and E_2_ further confirmed that supra-physiological E_2_ levels contributed to the immunological alterations. Collectively, further research is required to focus more on the environmental factors during the early stage of embryo development and subsequent specific effects such as epigenetic programming.

Our study is the first to evaluate peripheral immune profiles in ART-conceived children, including subgroup analysis of different embryo transfer and fertilization strategies. We still had several limitations. Firstly, this study is exploratory with a relatively small sample size, and further studies with a larger sample size are warranted. Secondly, the inclusion of twins may be more generalizable due to the high incidence of multiple pregnancies resulting from assisted reproductive technologies. Although we included only one of the twins, we cannot ignore the confounding bias associated with twins. Additionally, frozen embryo transfer consists of both natural cycle protocols and hormone replacement protocols, and we did not distinguish between these two protocols in this study. However, these two regimens may lead to different endocrine profiles with potential effects on the immunity of the offspring. Last but not least, we could not eliminate the impact of parental factors potentially influencing genetic propensity towards alterations in the immune response. Among these factors, infertility might be the most significant contributor to the differences in immunological markers, but we cannot rule it out. Future studies may be needed to comprehensively distinguish between the role of ART treatment and parental factors on immune indicators in offspring.

## Conclusion

Children born after fresh embryo transfer via ART displayed a higher risk of immune dysfunction in childhood manifested as elevated plasma IFN-γ and IL-4 levels and decreased ratio of IFN-γ to IL-4. The underlying mechanism is still unknown; exposure to supra-physical E_2_ levels might alter IL-4 levels. The elevated circulating IFN-γand IL-4 levels support the hypothesis that these children may face higher metabolic disorders and allergy disease risks in later life. Our study provided new insights into the effects of ART on the immune systems of offspring, which is of importance at the individual level and for the whole of society. Continuous monitoring and early intervention should be fully considered for these ART-conceived children.

## Supplementary Information

**Additional file 1 **: **Figure S1**.

## Data Availability

Not applicable.

## Abbreviations

ART: Assisted reproductive technology
NC: Naturally conceived
IFN-γ: Interferon-γ
IL: Interleukin
IVF: In vitro fertilization
ICSI: Intra-cytoplasmic sperm injection
Estradiol: E_2_
NK: Natural killer
T_H_: T helper
T_reg_: Regulatory T
BMI: Body mass index
PBMC: Peripheral blood mononuclear cell
PBS: Phosphate buffered saline
Gn: Gonadotropin
