# Supplementary figures and images for: The altered immunological status of children conceived by assisted reproductive technology
Source: Reprod Biol Endocrinol. 2021 Nov 26;19:171. doi: 10.1186/s12958-021-00858-2 (PMC8620159; doi:10.1186/s12958-021-00858-2)

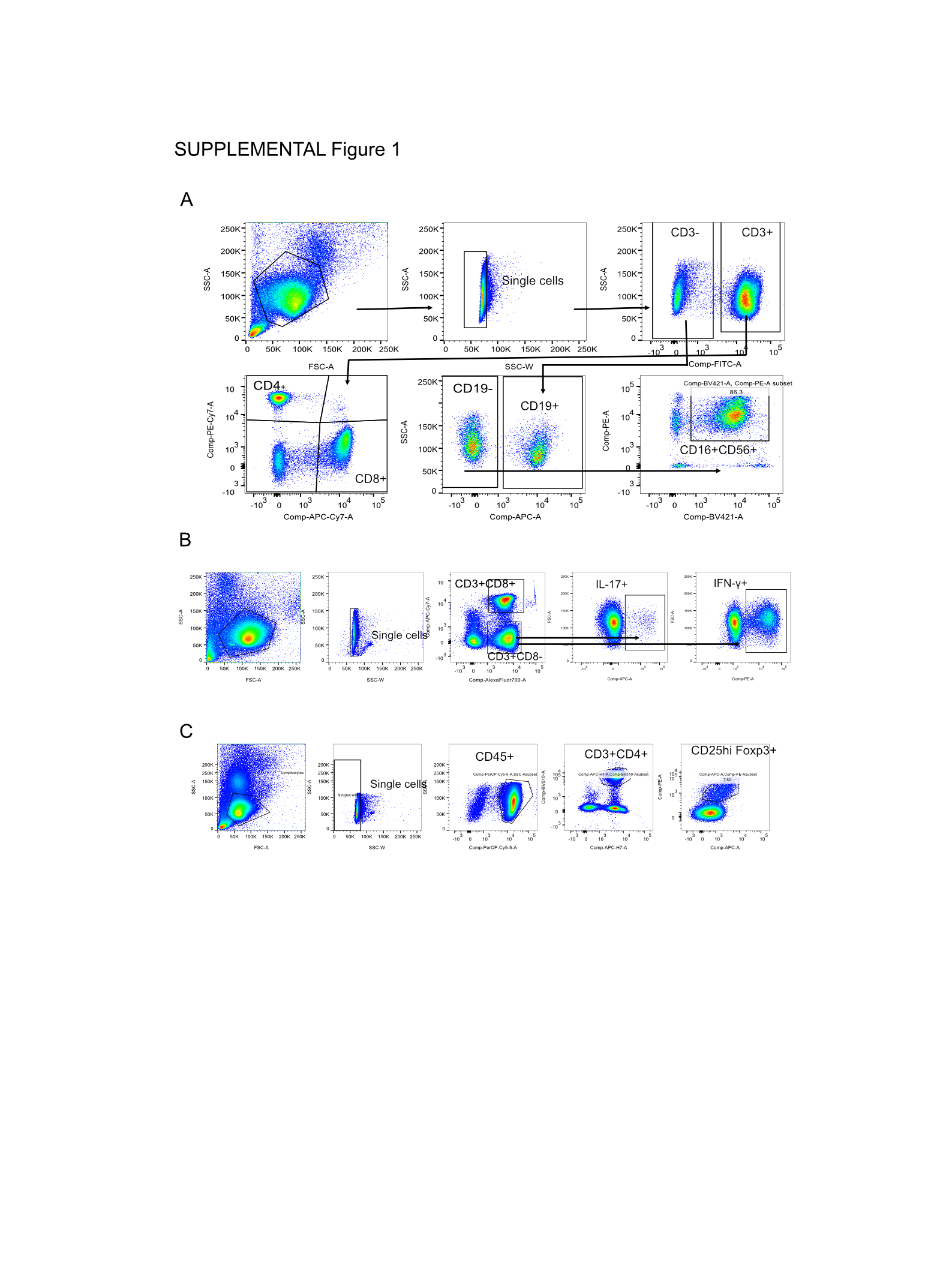

Supplement: Supplementary file 1 — Additional file 1 : Figure S1. [file 12958_2021_858_MOESM1_ESM.tiff]
